# Needle loss in subclavian vein during central venous catheter placement: case report of a rare complication

**DOI:** 10.1186/s13037-014-0049-y

**Published:** 2015-02-24

**Authors:** Daniela Botolin, Annie Mooser, Duane Stillions, Keith Mortman, Shawn Sarin, Joseph Babrowicz

**Affiliations:** Department of Surgery, The George Washington University Hospital, 2150 Pennsylvania Avenue, NW, Suite 6B, Washington, DC 20037 USA; Division of General Surgery, Walter Reed National Military Medical Center, 8901 Rockville Pike, Arrowhead Building, 1st Floor, Bethesda, MD 20889 USA; Department of Anesthesiology and Critical Care Medicine, The George Washington University Hospital, 900 23rd Street, NW, Washington, DC 20037 USA; Division of Thoracic Surgery, The George Washington University Hospital, Foggy Bottom South Pavilion, 22nd & I Street, NW, 6th Floor, Washington, DC 20037 USA; Department of Radiology, The George Washington University Hospital, 900 23rd Street, NW, Washington, DC 20037 USA; Division of Vascular Surgery, The George Washington University Hospital, Foggy Bottom South Pavilion, 22nd & I Street, NW, 6th Floor, Washington, DC 20037 USA

**Keywords:** Subclavian, Catheter, Fluoroscopy, Ultrasound, Obesity

## Abstract

We present a case of needle separation during central venous catheter (CVC) placement in a super morbidly obese patient with subsequent surgical intervention in its retrieval. This complication, potentially lethal due to the relevant anatomy of such a procedure, alerts critical care physicians and surgeons to the possibility of equipment failure and stresses proper technique in what has become a routine procedure. It also emphasizes the routine use of ultrasound-guidance for cannulation in patients of any body habitus. While infection and arrhythmia are the generally known complications of CVC placement, clinicians must be alert to unanticipated events such as needle separation. In our case, the retrieval of this needle required multi-disciplinary intervention between radiology, critical care, vascular surgery, and thoracic surgery. Our event stresses hypervigilance to complications in a common procedure.

## Background

Within the critical care setting, CVCs are commonly used for intravenous therapy, dialysis, and the delivery of medications and nutrition, among other clinical interventions. CVC use was first documented by Werner Forssman, who as a surgical intern used his own cephalic vein to canalize his right atrium in 1929. In 1953, Seldinger perfected this technique [1]. In 1998, physicians in the United States inserted more than 5 million CVCs [2]. In 2011, 15 million catheter days, defined as the total number of days of patient exposure to CVCs, were documented in intensive care units across the country [3].

As with any procedure, proper precautions must be taken to avoid complications of CVC placement. With regard to catheters, this often emphasizes infection risk. There are complications of mechanical nature, as well. We report a rare event of needle separation during CVC insertion in the subclavian vein and the complexity of its removal.

## Case presentation

A 46-year-old super morbidly obese male (BMI: 72) in renal failure requiring hemodialysis was admitted to the intensive care unit (ICU) following herniorrhaphy for an acute ventral hernia and incarcerated transverse colon. On post-operative day #2, CVC placement into his left subclavian vein with an 18-gauge access needle of 6.35cm length was attempted at the bedside in the intensive care unit. Due to the patient’s excessive body mass it was necessary to use the entire length of the needle.

Following successful venous puncture with minimally applied pressure, the 18-gauge access needle fractured and separated from the hub device. At this time it was unknown if the foreign body was in the vascular space or in the left chest wall.

Initial attempt at retrieval took place at the bedside. The incision site was immediately extended in an attempt to track and retrieve the needle, which was palpated running parallel to the clavicle. Given the proximity to the great vessels and poor visualization, bedside intervention was stopped.

**Figure 1:**
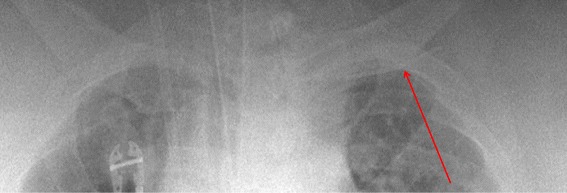
**Portable AP Chest X-ray showing the fractured access needle within the left anterior chest wall.**

A portable AP chest x-ray taken just after needle fracture showed a curvilinear density inferior to the left clavicle (Figure 1).

**Figure 2:**
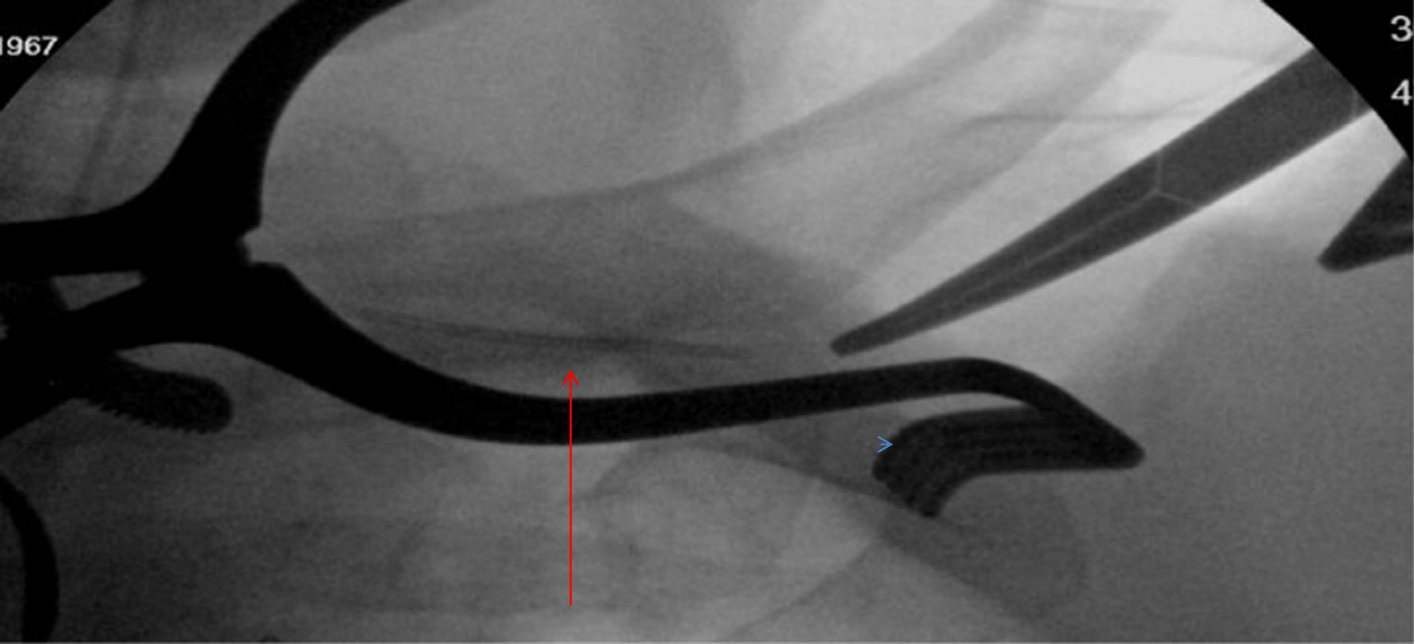
**Intraoperative tonsil clamp and fluoroscopy indicated the needle positioned medial to the bedside incision, deep to the costoclavicular ligament.**

On post-operative day #3 the patient was taken to the operating room for needle retrieval under fluoroscopic guidance. The needle was visualized medial and deep to the previous chest wall incision. Muscles of the chest wall were divided and mobilized to expose the clavicle. Repeat fluoroscopy revealed the needle to be deep and medial to the costoclavicular ligament, approaching the chest (Figure 2).

To approximate the needle, the costoclavicular ligament was divided, and the subclavian vein was exposed. The original puncture site was encountered in the middle segment of the subclavian vein. Using this reference point, oblique images on the c-arm were then taken, and the retained needle was seen deep to the clavicle, still in the subclavian vein.

**Figure 3:**
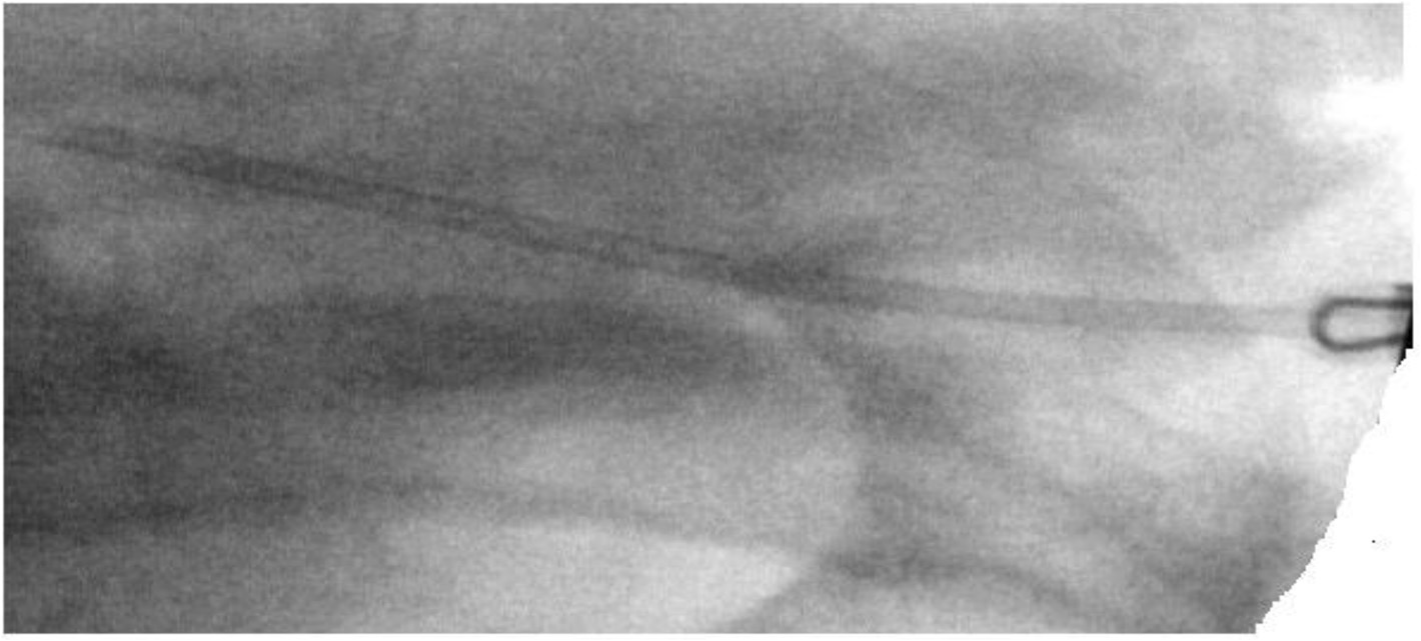
**A micropuncture access set was used to puncture through the soft tissues on the lateral pectoral region into a thoraco-acromial vein branch which had been exposed laterally on the subclavian vein.** A snare device was passed through an 8-French sheath to engage and remove the needle.

An endovascular approach was then used under fluoroscopy to retrieve the needle through the thoracoacromial vein. Ultimately, a snare device engaged the needle. The needle and snare were controlled with a sheath, and the needle was retracted successfully in one unit through the venous thoracoacromial branch (Figure 3).

## Conclusions

Clinicians must be familiar with both the frequent and uncommon complications of this often used instrumentation. These complications have been divided into infectious, mechanical, and thrombotic categories [4]. Our event was mechanical in nature. According to past reviews, mechanical complications are more often associated with subclavian-site placement as compared to the internal jugular vein or femoral vein [5,6]. Specifically, compared to internal jugular CVC (35.9% mechanical complication rate), subclavian sight complications are slightly higher at 39%. Of these, arterial puncture, hematoma, and pneumothorax are most common [7]. There is no previous documentation to our knowledge of needle fracture at either an internal jugular, subclavian, or femoral site.

Unlike the technical error often associated with loss of the guide wire during catheterization [8] we attribute the access needle fracture to a faulty instrument. There was no deviation from standard technique; however, given the patient’s body mass it is possible that our needle bent from overlying pressure of the soft tissue without excessive applied force.

With regard to visualization during attempted access, we did not use ultrasound. In critical care patients, real-time ultrasound has been acknowledged for lower incidence of mechanical complications compared to the landmark method [9]. We were confident in infra-clavicular positioning as an appropriate guide despite the patient’s significant BMI. As stated above, we were able to palpate the needle superficially through the tissues after its initial detachment from the hub. Despite our confidence in positioning, retrospective discussion of our case highlights the importance of ultra-sound guidance in subclavian vein catheterization. Real-time ultrasound may have provided visual documentation of the needle bending under the stress of the patient’s tissues prior to its departure from the hub.

While the consequences of needle fracture and loss within the vascular space have the potential for fatal complication, we were able to avoid hemodynamic compromise and lung injury by limiting our attempts for needle retrieval at the bedside. Our continued patient monitoring in the critical care setting and repeat chest x-ray ensured the stability of the fractured needle until operative intervention. The operating room is the optimal environment for intervention to retrieve an intravascular retained foreign body with such proximity to the great vessels.

Our case stresses that real-time ultrasound guidance in patients of any body habitus, including the super morbidly obese, should be standard of care. Technical difficulties include accurate probe placement and excessive infra-clavicular tissue obscuring angle of penetration. However, a clear two-dimensional image of the subclavian vein and overlying tissues may have provided visualization of the needle bending prior to this event and avoided necessity for such complicated retrieval.

## Consent

Written informed consent was obtained from the patient’s next of kin for publication of this case report and any accompanying images. A copy of the written consent is available for review by the Editor-in-Chief of this journal.

## Abbreviations

CVC: Central venous catheter
BMI: Body Mass index
ICU: Intensive care unit
AP: Anterior-Posterior
